# Effect of cobalt addition on the structure and properties of Ni–MCM-41 for the partial oxidation of methane to syngas

**DOI:** 10.1039/c9ra03534f

**Published:** 2019-08-15

**Authors:** Yufeng Li, Junwen Wang, Chuanmin Ding, Lichao Ma, Yanan Xue, Jing Guo, Shunqiang Wang, Yuanyuan Meng, Kan Zhang, Ping Liu

**Affiliations:** College of Chemistry & Chemical Engineering, Taiyuan University of Technology Taiyuan 030024 PR China wangjunwen@tyut.edu.cn dingchuanmin@tyut.edu.cn +86 351 6014 498 +86 351 6014 498; Institute of Coal Chemistry, Chinese Academy of Sciences Taiyuan 030001 PR China

## Abstract

A one-step hydrothermal crystallization method was used to synthesize Co–Ni–MCM-41 catalysts for the partial oxidation of methane to syngas reaction. Co was added as an assistant in the synthesis process. The formation of a Ni–Co alloy decreased the damage of Ni ions to the framework of MCM-41. The Ni–Co alloy introduced more Ni into the channel exposing more active sites. The properties of the synthesized catalysts were characterized by XRD, N_2_ adsorption–desorption, TEM, ICP, FT-IR, H_2_-TPR, XPS and TGA techniques. Co–Ni–MCM-41 catalysts showed superior catalytic performance and sintering resistance than Ni–MCM-41 catalyst without Co. The Ni–Co alloy inhibited the formation of the NiO, thus reducing the sintering of the catalyst. The result was attributed to higher metal dispersion and more regular pore structure of the Co–Ni–MCM-41 catalysts. When the Co content was 1%, a conversion of 88% and selectivity of 87% was achieved.

## Abstract 2

The Ni–Co alloy confined within MCM-41 improved dispersibility and the stability of Ni.

## Graphical abstract

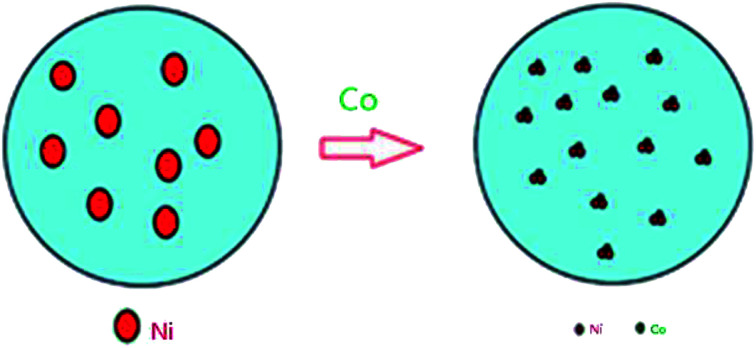

## 1. Introduction

In recent years, methane (CH_4_) has attracted enormous attention as an efficient and clean energy source. The partial oxidation of methane reaction (POM) provides an important intermediate (syngas) for chemical processes.^1,2^ Compared with conventional steam methane reforming (CSMR), POM is a mild exothermic reaction with a rapid reaction rate, high CH_4_ conversion and requires a smaller reactor. In addition, the suitable H_2_/CO ratio of 2 is beneficial to methanol synthesis and Fischer–Tropsch synthesis.^3,4^

Ni-based catalysts have been the subject of many POM research studies due to their excellent performance, high abundance and low cost.^5^ However, Ni-based catalysts have the problems of deactivation due to carbon deposition and sintering at high temperatures.^6^ Much effort has been made to improve the anti-carbon and anti-sintering properties of the Ni-based catalysts, such as increasing metal dispersion, decreasing the metal particle size, confining the active components within porous materials.^7–9^ The confinement of the zeolite or mesoporous materials can effectively control the agglomeration and sintering of the nanoparticles. Iglesia developed a strategy to encapsulate a series of metal clusters (Pt, Pd, Ru, and Rh) in different aluminosilicate zeolites such as SOD, GIS, ANA and LTA zeolites.^10–12^ The confinement effect of zeolite improved the catalytic performance and stability of the catalyst. However, the small pores and the acidity of these zeolites are not conducive to the POM reaction.

The MCM-41 mesoporous molecular sieve has been widely studied for its high specific surface area, regular pore structure and high thermal stability.^13^ The ordered mesoporous structure is more favorable for POM reaction compared with the microporous molecular sieve. Nickel has been successfully encapsulated in the MCM-41 mesoporous materials.^14,15^ However, a large amount of nickel entered the framework, leading to the destruction of the molecular sieve structure and the reduction of the active sites.

Previous studies have shown that the addition of additives can effectively improve the catalytic performance of nickel-based catalysts, prevent the loss of Ni nanoparticles and inhibit the formation of carbon deposition.^16–18^ A CoNi@SiO_2_ catalyst was prepared by Li and applied in the POM reaction.^19^ Compared with Ni@SiO_2_ and Co@SiO_2_ catalysts, it showed better catalytic activity and anti-carbon deposition performance due to the formation of a Co–Ni alloy. The alloy improved the reduction temperature of the catalyst thus inhibiting carbon deposition over the catalyst at high temperatures.

The Ni–Co alloy confined within MCM-41 may improve the dispersibility of Ni and avoid destruction of the molecular sieve structure. At the same time, the alloy may be beneficial to enhance the stability of nickel. Herein, the MCM-41 zeolite was used to encapsulate metallic nickel and cobalt under direct hydrothermal conditions. The role of introduced Co on catalytic performance and stability was investigated further.

## 2. Experimental section

### 2.1 Catalyst preparation

The Co–Ni–MCM-41 catalysts were synthesized *via* one-step hydrothermal crystallization method.^14^ Cetyltrimethylammonium bromide (CTAB) was used as the structural template and tetraethylorthosilicate (TEOS) was used as silicon source. Typically, 2.345 g CTAB was dissolved in the 100 mL distilled water and stirred for 30 min at ambient temperature. Appropriate amount of Ni(NO_3_)_2_·6H_2_O was dissolved in 20 mL distilled water. Ultrasonic oscillation was conducted for 5 min until the Ni(NO_3_)_2_·6H_2_O was fully dissolved and ammonia solution was added slowly to obtain a complex Ni(NH_3_)_6_^2+^ solution with pH of 10. Then the two solutions were mixed and stirred for 30 min. 10 mL TEOS was dropped into the mixture and ammonia solution was used to adjust the pH of the solution to 10. The molar ratio of the composition was 1.0SiO_2_ : 0.152CTAB : 2.8NH_3_ : 0.1Ni : *x*Co : 141.2H_2_O (*x* = 0.005, 0.01, 0.015). After continuous stirring for 6 h, the mixture was transferred into a Teflon-lined stainless steel autoclave and crystallized at 110 °C for 48 h. The resulting solid was filtered, washed with deionized water until neutral, dried for 24 h at 70 °C and finally calcined at 550 °C for 6 h. The obtained catalysts were denoted as 0.5Co–Ni–MCM-41, 1Co–Ni–MCM-41 and 1.5Co–Ni–MCM-41. Additionally, Ni–MCM-41 and Co–MCM-41 was prepared by similar method.

### 2.2 Catalyst characterization

The as-prepared catalyst was characterized by X-ray diffraction using a BRUKER AXS D8 ADVANCE diffractometer with Cu Kα radiation (40 kV, 40 mA) at a scanning rate of 2° min^−1^ in both small angle (2*θ* range 0.5–10°) and wide angle (2*θ* range 10–80°). N_2_ adsorption–desorption isotherms were measured on a Micrometrics ASPA 2000 gas adsorption analyzer. Before test, the sample was degassed under high vacuum at 200 °C for 12 h. The TEM images were taken over a Tecnai G2-F30 instrument. The content of active components of the catalyst was determined by Thermo ICAP6300 inductively coupled plasma atomic emission spectrometer. The framework of the catalyst was measured using a Nicolet-Impact 400 FT-IR spectrometer. Temperature programmed reduction of H_2_ (H_2_-TPR) was performed on a chemical adsorption analyzer (TP-5676) equipped with a thermal conductive detector to study the reducibility of the catalyst. 50 mg samples were heated to 400 °C at a rate of 10 °C min^−1^ under N_2_ flow of 50 mL min^−1^ and kept at this temperature for 1 h to remove adsorbed water. After cooled down to room temperature, the samples were switched to a 25% H_2_/N_2_ (v/v, 60 mL min^−1^) mixture. The sample temperature was programmed to 900 °C at rate of 10 °C min^−1^. The surface oxidation states of Ni and Co were analyzed using X-ray photoelectron spectroscopy (XPS) on a Thermo ESCALAB 250 spectrometer. TG data was recorded on a SETARAM thermal analyzer in a 50 mL min^−1^ air flow from room temperature to 1000 °C with heating rate of 10 °C min^−1^.

### 2.3 Catalyst evaluation

The catalytic activity was tested in the fixed bed quartz tubular reactor (inner diameter: 10 mm) at atmospheric pressure. 0.5 g catalyst sandwiched by two silica wool was placed in the center of the quartz tube. A thermocouple fixed in the furnace was used to monitor and measure the temperature of the catalyst bed. Before the reaction, the catalysts were reduced by H_2_ with a flow rate of 70 mL min^−1^ at 750 °C for 2 h. Then CH_4_ and O_2_ (2 : 1, molar ratio) with a total flow of 150 mL min^−1^ (GHSV = 18 L g^−1^ h^−1^) were fed into the reactor. The catalysts were tested in the temperature of 750 °C. The catalytic activity tests of different GHSV (10.8–32.4 L g^−1^ h^−1^) were operated at 750 °C. The outlet mixture products were analyzed by a GC-920 gas chromatograph equipped with TCD and FID detectors.

In this work, methane conversion (*X*_CH_4__), CO selectivity (*S*_CO_) and H_2_ selectivity (*S*_H_2__) were investigated to determine the catalytic performance of the catalysts. All the performance data of catalysts were calculated using the following equations:1
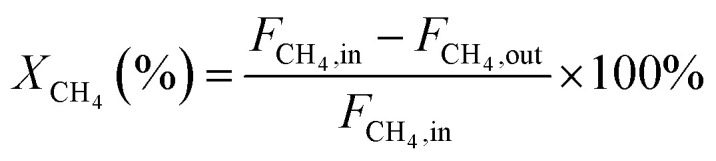
2
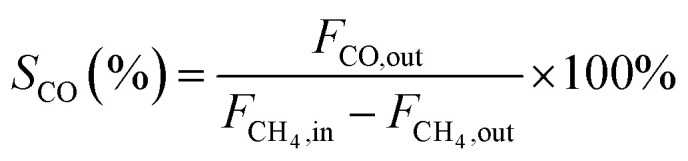
3
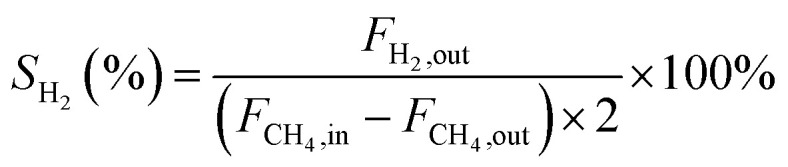
In these equations, *F*_CH_4_,in_, *F*_CH_4_,out_ denotes the CH_4_ mole of inlet gas and CH_4_ mole of outlet gas; *F*_CO,out_, *F*_H_2_,out_ denotes the CO and H_2_ mole of outlet gas, respectively.

## 3. Results and discussion

### 3.1 Characterization

#### 3.1.1 Phase composition

**Fig. 1:**
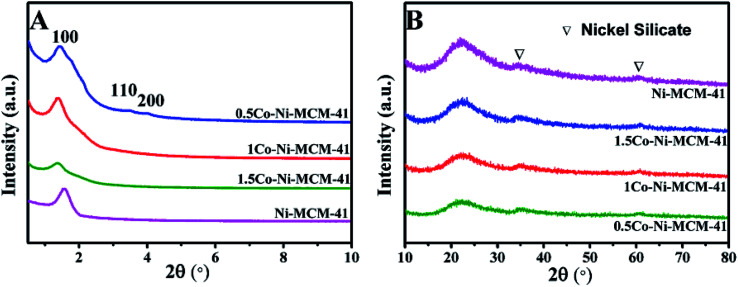
Small angle XRD patterns (A) and wide angle XRD patterns (B) of Ni–MCM-41 and Co–Ni–MCM-41 catalysts.

Fig. 1A showed the small angle XRD of the as-prepared catalysts. All the catalyst had a strong diffraction peaks between 1 and 3° and two weak diffractions between 3 and 6° which correspond to (100), (110), (200) lattice planes of the MCM-41, respectively.^20^ The result showed that all the catalysts possessed the long-range ordered hexagonal mesoporous framework of the MCM-41. Compared with the Ni–MCM-41 catalysts, the diffraction peaks of the Co–Ni–MCM-41 catalysts shifted to low angles, which indicated that Co entered the inside of the molecular sieve.^21^ The diffraction peak intensity of (100) lattice decreased and widened gradually as the Co content increased. Although the molecular sieve retained the hexagon mesoporous framework, the entry of Co ions partly destroyed the framework structure and affected the long-range order. In addition, compared with Ni–MCM-41, the 0.5Co–Ni–MCM-41 showed more regular pore structure. It might be due to the formation of Ni–Co alloy reduced the damage of Ni ions to the framework.

Fig. 1B showed the wide angle XRD of the as-prepared catalysts. There were no obvious diffraction peaks of metal species from the XRD patterns, indicating that most metal ions were inserted into the framework or highly dispersed in the channel of the molecular sieve. It was worth noting that nickel silicate reflections of 0.5Co–Ni–MCM-41 was lower than those of Ni–MCM-41, which suggested that there were less nickel silicate existing in 0.5Co–Ni–MCM-41.^22^ This may be due to that a part of Ni ions formed alloy with Co ions, which reduced the content of Ni ions connected to the framework. The results were in accord with the small angle XRD analysis.

**Fig. 2:**
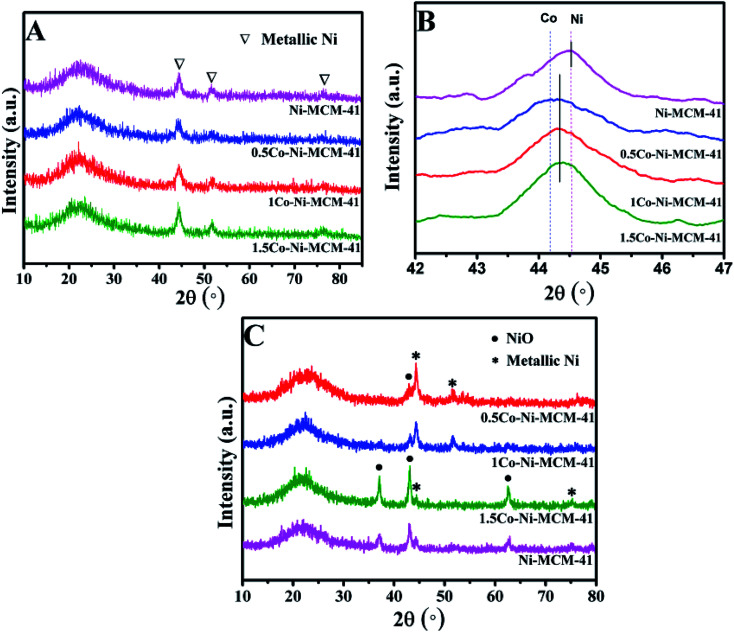
Wide angle XRD patterns of reduced catalysts (A and B) and used catalysts (C).

The diffraction patterns of the reduced catalysts were displayed in Fig. 2A and B. The diffraction peaks of Ni phase could be observed in all the reduced catalysts. Fig. 2B presented the XRD analysis of the reduced catalysts in the range of 2 theta between 42° and 47°. The diffraction peak at 2 theta of 44.52° in Ni–MCM-41 was assigned to Ni (File no. 04-0850). With the addition of Co, it could be seen that the maxima of the metallic peak were shifted to low angle (44.38°) while the diffraction peak of Co at 44.22° (File no. 15-806) was absent. Occurrence of the new peak at 44.38° indicated the formation of a Ni–Co alloy in Co–Ni–MCM-41 catalysts. Similar conclusions were reported over different support materials in the previous studies.^19,23,24^ Takanabe *et al.*^25^ found that the diffraction peak at 2 theta of 44.22° (Co) were shifted to 44.51° (Ni) as the Co : Ni increased from 0 : 100 to 100 : 0 in the titania supported bimetallic catalysts. XRD patterns of SBA-15 loaded with Ni and Co catalysts also supported this conclusion.^26^ In addition, these studies proved the Ni–Co alloy in bimetallic catalysts suppressed the oxidation of cobalt.^27,28^ The formation of Ni–Co alloy was further discussed in the following sections. The low diffraction peak of metallic particles in the 0.5Co–Ni–MCM-41 catalyst indicated that the smaller metal particles were well dispersed in the catalyst. With the increasing of Co content, the diffraction peak intensity of the metal gradually increased, accompanied by the narrowing of the half-peak width, which indicating the growth of the metallic particles. It might be the fact that the formation of Ni–Co alloy decreased the Ni species entering the framework. The interaction between metal Ni and molecular sieve was weakened, leading to the metal agglomeration at high temperature. In addition, it was found that the influence on the size of Ni particles was small when the content of Co was less than 1% according to Debye–Scherrer formula.^29^

Fig. 2C showed the diffraction patterns of the used catalysts. The 0.5Co–Ni–MCM-41 and 1Co–Ni–MCM-41 exhibited high reflection of metallic Ni, while most Ni existed as oxides in the Ni–MCM-41 and 1.5Co–Ni–MCM-41. This result might be due to the formation of Ni–Co alloy inhibited the oxidation of Ni in the Co–Ni–MCM-41 catalysts. In addition, the NiO particle size in 1.5Co–Ni–MCM-41 catalyst was larger that of other catalysts. It might be due to that the addition of a large number of Co ions caused the collapse of the framework of the molecular sieve, Ni particles were more likely to form NiO particles with oxygen without the confinement of the channel and bare NiO particles were easily aggregated into larger particles.^30^

#### 3.1.2 Skeletal structure analysis with FT-IR spectra

**Fig. 3:**
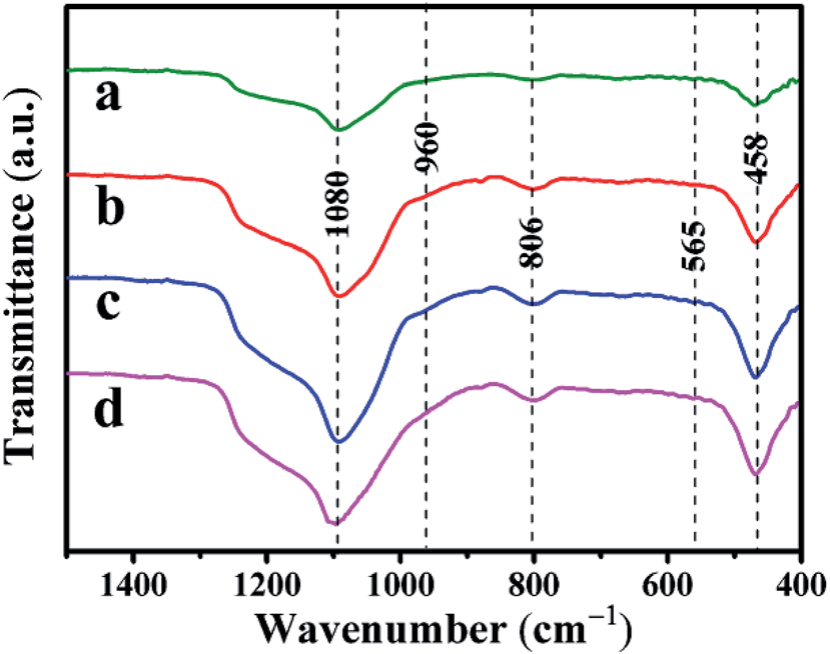
FT-IR spectra of the catalysts: (a) 0.5Co–Ni–MCM-41 (b) 1Co–Ni–MCM-41 (c) 1.5Co–Ni–MCM-41 (d) Ni–MCM-41.

The FT-IR spectra of the Co–Ni–MCM-41 and Ni–MCM-41 catalysts were presented in Fig. 3. The absorption bands at 1080 cm^−1^ and 806 cm^−1^ were the asymmetric and symmetric stretch of the Si–O–Si bridges, respectively.^31^ The bands at 458 cm^−1^ were due to Si–O bending vibration and the bands at 565 cm^−1^ were assigned to the symmetric stretching vibration of Si–O–Si bridges.^32^ The shoulder peak at 960 cm^−1^ was used to prove that the metal entered the framework of the molecular sieve, thus weakening the vibration of the Si–O bond.^33^ It could be seen that the vibration intensity of all the catalysts at 960 cm^−1^ had hardly changed, indicating that the catalyst framework still interacted with Ni strongly.

#### 3.1.3 N_2_ adsorption–desorption analysis

**Fig. 4:**
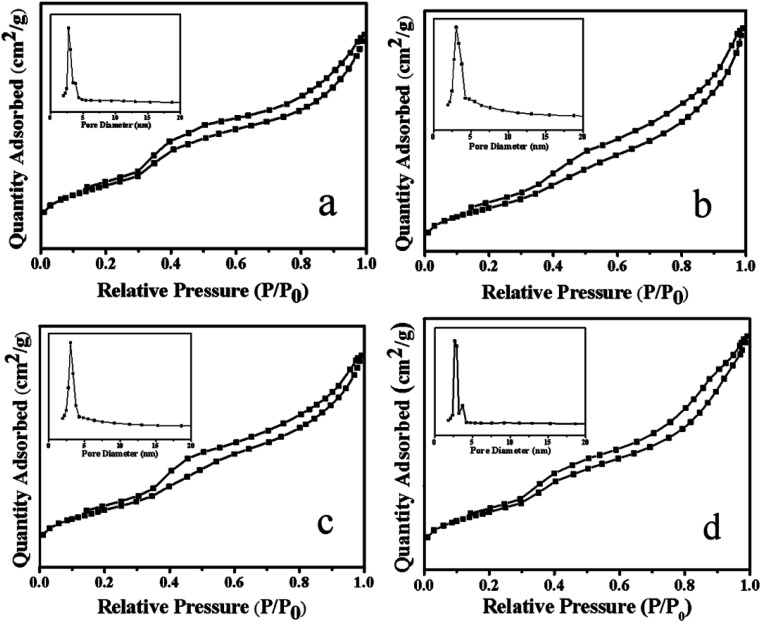
Adsorption–desorption isotherms and pore size distribution of the catalysts: (a) 0.5Co–Ni–MCM-41 (b) 1Co–Ni–MCM-41 (c) 1.5Co–Ni–MCM-41 (d) Ni–MCM-41.

Fig. 4 showed the N_2_ adsorption–desorption curve and pore size distribution of the Ni–MCM-41 and the Co–Ni–MCM-41 catalysts. All the catalysts exhibited the type IV isothermal curves and type H1 hysteresis loops, indicated typic characteristic of the mesoporous materials.^34^ The hysteresis loop between the relative pressure of 0.8 and 1.0 indicated that the catalysts possessed mesoporous structure due to mesoporous stacking. Comparing the adsorption and desorption curves of Ni–MCM-41 and Co–Ni–MCM-41 catalysts, it could be found that with the increase of Co content in the catalyst, the H1 type hysteresis ring gradually became larger, which proved that more mesoporous structures were formed. The reason was that the formation of Ni–Co alloy inhibited the connection between Ni and the framework of molecular, thus protecting the original well-ordered mesoporous structure of the MCM-41. It was observed that the pore size of the 0.5Co–Ni–MCM-41 catalyst was smaller than that of the Ni–MCM-41 catalyst. However, the pore size increased with the increase of Co. The reason might be that more Ni–Co alloy entered into the molecular sieve channel, resulting in the increase of pore size, and the increase of pore size could somewhat indicate the damage of Co to the channel. With the increased of Co content, the specific surface area pore volume of the Co–Ni–MCM-41 catalysts gradually decreased (Table 1), while the pore diameter gradually increased due to the pore blockage caused by Ni–Co alloy entering into the channel of the molecular sieve.

**Table 1:** Characteristics of the catalysts determined by N_2_ sorption and ICP

Catalysts	Ni^a^ (wt%)	Ni–NH_4_AC^a^ (wt%)	Surface area^b^ (m^2^ g^−1^)	Pore volume^c^ (cm^3^ g^−1^)	Pore size^d^ (nm)
Ni–MCM-41	8.92	5.74	594	0.69	4.77
0.5Co–Ni–MCM-41	9.01	5.48	541	0.74	4.68
1Co–Ni–MCM-41	8.87	5.26	472	0.68	4.99
1.5Co–Ni–MCM-41	9.12	5.02	382	0.63	5.57

a Calculated by ICP.

b Calculated by the BET.

c BJH desorption pore volume.

d BJH desorption average pore size.

#### 3.1.4 Microstructure of the catalysts

**Fig. 5:**
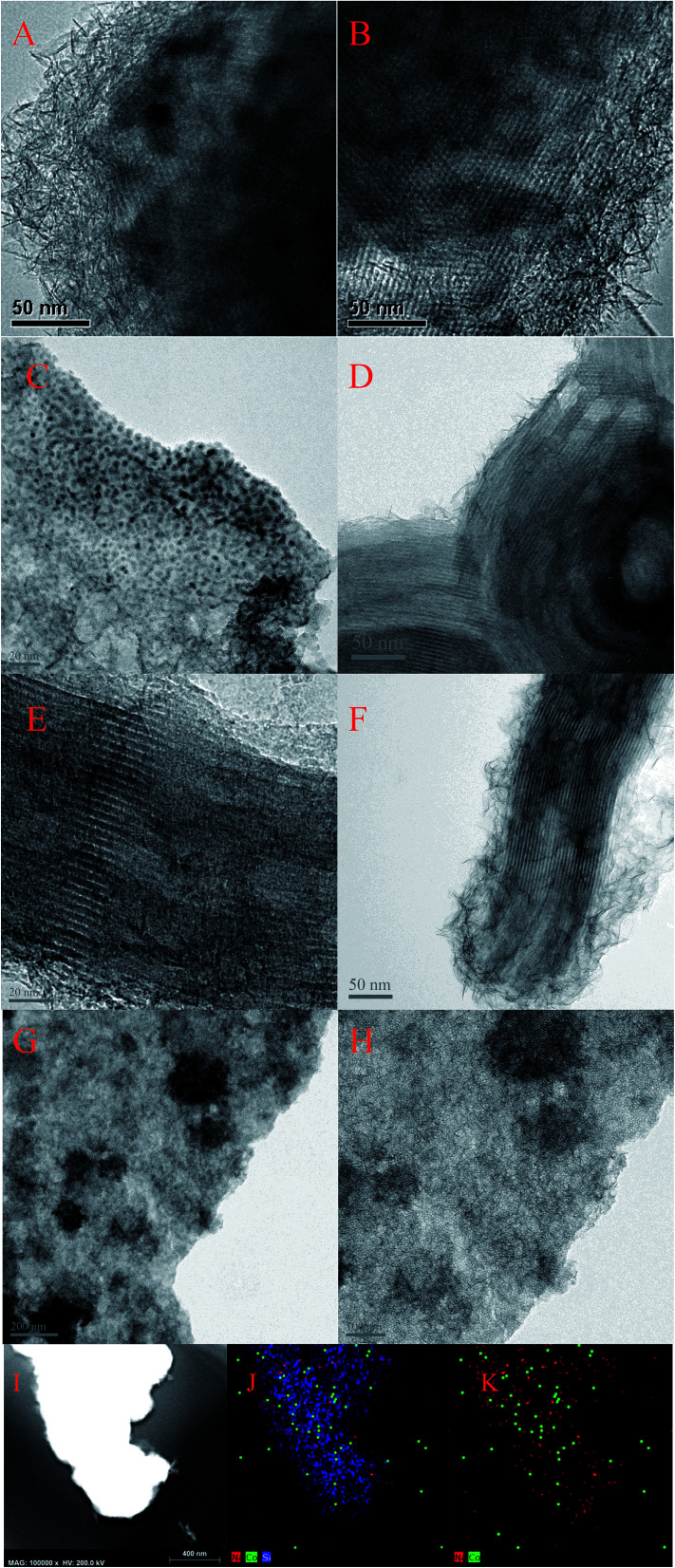
TEM image of fresh catalysts: (A and B) Ni–MCM-41 (C and D) 0.5Co–Ni–MCM-41 (E and F) 1Co–Ni–MCM-41 (G and H) 1.5Co–Ni–MCM-41 catalysts with EDS elemental mapping of the 1Co–Ni–MCM-41 catalyst (I–K).

Fig. 5 showed the morphologies of Ni–MCM-41 and Co–Ni–MCM-41 catalysts. Long-range ordered channel structure was observed in Ni–MCM-41, 0.5Co–Ni–MCM-41 and 1Co–Ni–MCM-41 catalysts (Fig. 5B, D and F), and 0.5Co–Ni–MCM-41 presented more regular channels than that of Ni–MCM-41. This result was accord to the small angle XRD analysis. However, Fig. 5G and H showed the collapse of the zeolite structure and the agglomeration of metal particles (∼200 nm). As the Co content increase, more silicon atoms were substituted for Co atoms, leading to the degradation of the pore system. In addition, small metal particles (∼1.5 nm) were observed in 0.5Co–Ni–MCM-41 (Fig. 5C) while there were few larger metal particles (12 nm) in Ni–MCM-41 catalyst. This result proved that the addition of Co was beneficial to the formation of metals in channels rather than in the silica framework.^14^ Small black spots which assigned to metal particles (<1 nm) were also observed in 1Co–MCM-41 catalyst (Fig. 5E). It could be seen that appropriate addition of Co could effectively increase the dispersion of Ni while the addition of large amounts of Co destroyed the framework of MCM-41, causing the metal aggregation without the confinement of the framework. Energy-dispersive spectroscopy (EDS) was used to observe the distribution of metals in MCM-41. As shown in Fig. 5I–K, Co element had similar distribution with Ni element. The results turned out that Co and Ni was uniformly distributed in the 1Co–Ni–MCM-41.

#### 3.1.5 Elemental analysis

In order to prove that the addition of Co ions could inhibit the connection between Ni ions and the framework of the molecular sieve, the as-prepared Co–Ni–MCM-41 catalyst was cleaned with ammonium acetate to remove Ni in the pore channel of the molecular sieve,^35,36^ and the content of Ni in the catalyst before and after cleaning (Ni–NH_4_AC) was tested by ICP. As shown in Table 1, with the increase of Co content, the Ni content in the skeleton gradually decreased from 5.74% to 5.02%, while the Ni content in the pore passage gradually increased from 3.18% to 4.1%. It was further proved that the addition of Co decreased the content of Ni in the skeleton to some extent and protected the skeleton structure of the molecular sieve.

#### 3.1.6 H_2_-TPR analysis

**Fig. 6:**
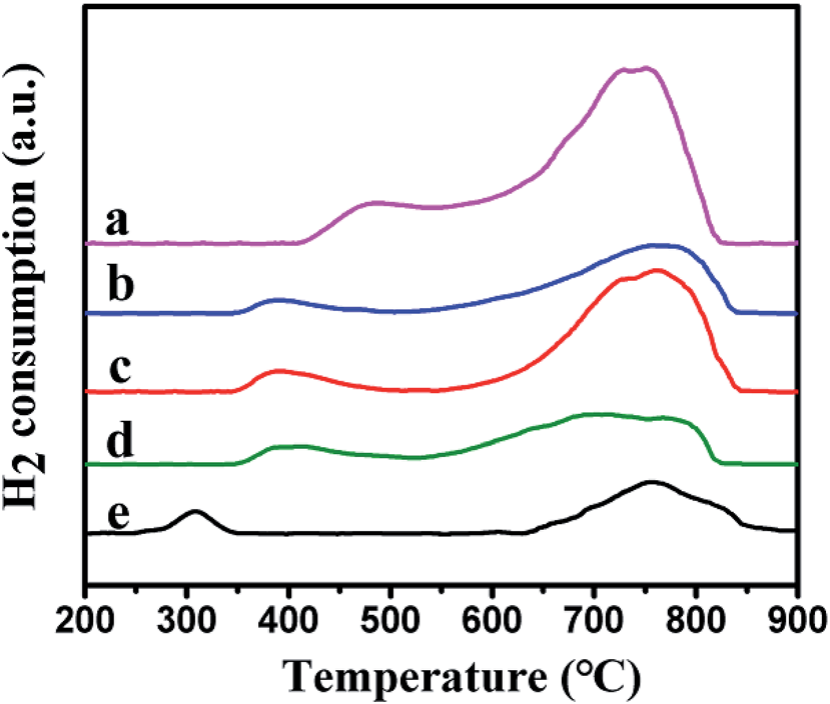
H_2_-TPR profiles of the catalysts: (a) Ni–MCM-41, (b) 0.5Co–Ni–MCM-41, (c) 1Co–Ni–MCM-41, (d) 1.5Co–Ni–MCM-41 and (e) Co–MCM-41.

The reduction behavior of the catalysts was tested by using H_2_-TPR. Fig. 6 showed the reduction curves of the different samples. Two reduction peaks were observed in the low temperature and the high temperature regions, respectively. For Ni–MCM-41 catalysts, the reduction peak at 500 °C were assigned to the reduction of bulk NiO and the higher temperature reduction peak at 750 °C were considered due to the strong interaction between small NiO particles and SiO_2_ framework.^22,37,38^ However, compared with the Ni–MCM-41 catalyst, the low temperature reduction peaks of the Co–Ni–MCM-41 catalysts were at around 380 °C, shifting to lower temperature area. To investigate this phenomenon, the H_2_-TPR profile of Co–MCM-41 (Co content was 1.5 wt%) was also presented in Fig. 6. A weak reduction peak around 310 °C which correspond to cobalt oxide species was observed. The single peak between those of Ni and Co oxides in Co–Ni–MCM-41 catalysts confirmed the formation of the Ni–Co alloy. Xu *et al.*^23^ found that the major reduction peak shifted to low temperature as Co component increase, indicating the existence of Co–Ni mixed oxide with homogeneity which was favorable for their reduction. The simultaneous reduction of Ni and Co was beneficial to the formation of Ni–Co alloy. Similar conclusions were also reported in other studies.^39,40^ In addition, Rynkowski *et al.*^41^ reported TPR of alumina-supported Ni–Pt samples and they found the single peak which assigned to one-stage reduction of bimetallic catalysts illustrated the alloying of metals in the reduction process. In our present study, one-step hydrothermal crystallization method was also thought to beneficial for the formation of Ni–Co alloy. The formation of Ni–Co alloy could weaken the interaction between the metal and support, and promote the reduction process.^42,43^

#### 3.1.7 Oxidation states of Ni and Co

**Fig. 7:**
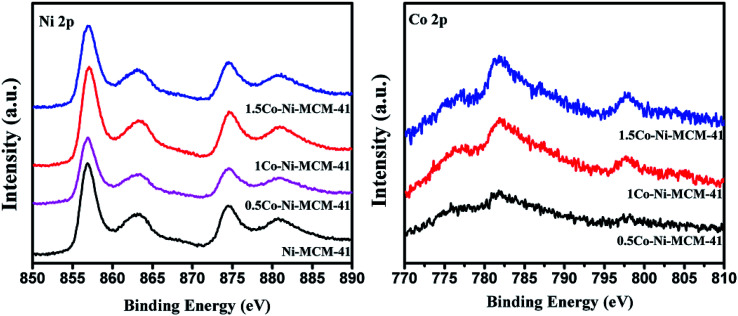
XPS spectra in the Ni 2p region and the Co 2p region of the as-prepared catalysts.

X-ray photoelectron spectroscopy (XPS) was used to investigate the oxidation of Ni and Co. Although XPS is known as a surface specific technique, the results could somewhat reflect the information of nanoclusters within the pores close to the surface of the zeolite crystal.^44^Fig. 7 showed the Ni 2p region of the as-prepared catalysts. It could be seen that there was an absent peak around 852.8 eV which assigned to metallic Ni. The peaks at 857.4 and 874.6 eV with satellite peaks at 863.6 and 881.1 eV was assigned to Ni^2+^ 2p_3/2_ and 2p_1/2_.^45^ The peaks appear at around 782 and 796 eV in the Co 2p region were ascribed to Co 2p_3/2_ and Co 2p_1/2_.^23,46^ However, there was no evidence of electronic effects arising from the alloying of Ni and Co, such as electron transfer between Ni and Co in Co–Ni–MCM-41 catalysts.^27^ Considering that we prepared Co–Ni–MCM-41 catalysts by one-step hydrothermal crystallization method rather than co-impregnation method, XPS could not fully reflect the information of the oxidation state of Ni and Co because Ni and Co might distribute deep in the catalysts.

#### 3.1.8 TGA analysis

**Fig. 8:**
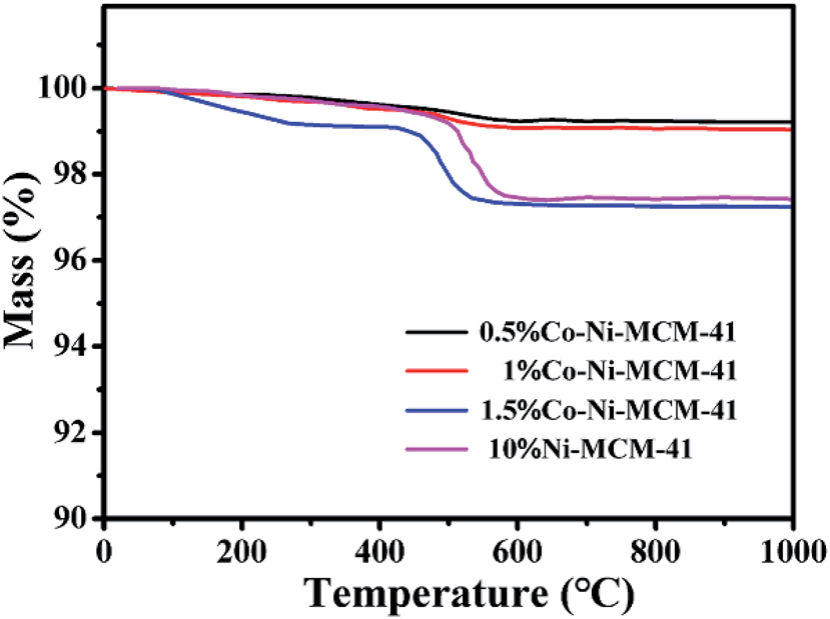
TGA of curves of the used catalysts.

Carbon deposition was an important index to evaluate POM reaction. Nickel-based catalysts were prone to carbon deposition at high temperature, which was one of the important reasons for catalyst deactivation. Fig. 8 showed the thermogravimetric diagram of the Co–Ni–MCM-41 and Ni–MCM-41 catalysts after reaction of 100 h at 750 °C. When the temperature reached about 450 °C, the carbon species on the surface of the catalyst were oxidized, resulting in the decline of the mass with carbon deposition.^43^ In addition, the mass of the Ni–MCM-41 and the 1.5Co–Ni–MCM-41 decreased significantly, indicating that the catalyst had more carbon deposition. There were some results showing the formation of the carbon deposition was related to oxidation species, which was accord with the XRD analysis of the used catalysts.^47^ The carbon deposition rate of the catalysts was 1.5Co–Ni–MCM-41 > Ni–MCM-41 > 1Co–Ni–MCM-41 ≈ 0.5Co–Ni–MCM-41, which proved that the addition of appropriate amount of Co could improve the anti-carbon deposition performance of the catalyst.

### 3.2 Catalytic performance of the catalysts

#### 3.2.1 Effect of Co and Co content on catalyst performance

**Fig. 9:**
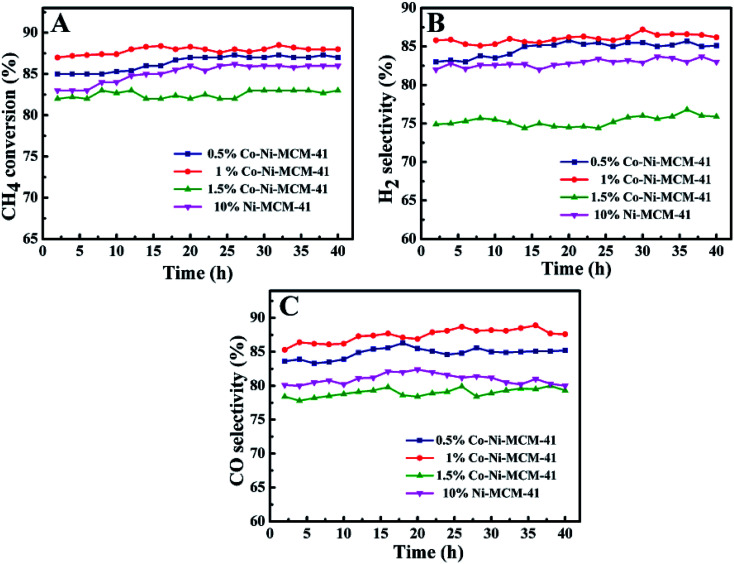
The catalytic ability comparison of the Ni–MCM-41 and Co–MCM-41 catalysts: (A) CH_4_ conversion, (B) H_2_ selectivity, (C) CO selectivity, at 750 °C, GHSV = 18 L g^−1^ h^−1^, atmospheric pressure.

Fig. 9 showed the CH_4_ conversion rate and H_2_, CO selectivity of the Co–Ni–MCM-41 and the Ni–MCM-41 catalysts in the POM reaction. The CH_4_ conversion and H_2_, CO selectivity of the 0.5Co–Ni–MCM-41 and 1Co–Ni–MCM-41 catalyst were both higher than that of the Ni–MCM-41 catalyst. The 1Co–Ni–MCM-41 catalyst showed excellent catalytic performance and displayed the highest CH_4_ conversion of 88%. According to the XRD analysis results after the reaction, the introduced Co ions were contributed to forming the Ni–Co alloy, which inhibited the Ni particles from combining with oxygen to form NiO particles, thus decreasing the sintering of the catalyst. It was worth noting that the catalytic activity of 1.5Co–Ni–MCM-41 catalyst was significantly lower than those of the other three catalysts, because the introduction of a large number of Co ions led to the collapse of the framework of the molecular sieve and the sharp decline in specific surface area. Part of the active sites in the catalyst were buried by SiO_2_, resulting in the reduction of catalytic activity.

Ni-based catalysts have been widely studied in methane reforming. Liu *et al.*^48^ prepared Ni-based bimetallic catalysts supported on MCM-41 by a direct hydrothermal method, and high metal dispersions were obtained with the incorporation of Zr cations, while remarkably low dispersions were obtained with the incorporation of Ti or Mn. Ni/ZrO_2_ catalyst prepared by hydrothermal method possessed small metal particles and higher catalytic performance (about 85% of CH_4_ conversion) in partial oxidation of methane.^49^ Habimana *et al.*^50^ reported Ni-based SBA-15 catalysts with Cu promoter for partial oxidation of methane to syngas, best catalytic performance was obtained in 12.5%Ni/2.5%Cu/SBA-15 catalyst (at 750 °C the conversion of CH_4_ reached 89%). When the Cu content was 10%, the catalytic performance was significantly affected (about 84% of CH_4_ conversion). In the present study, the appropriate addition of Co in the Ni–MCM-41 catalyst could increase the dispersion of the metals and prevent the oxidation of Ni which was contributed to the improvement of the catalytic activity. And our catalysts remained high active after reaction of 100 h with little carbon deposition.

#### 3.2.2 Catalytic stability

**Fig. 10:**
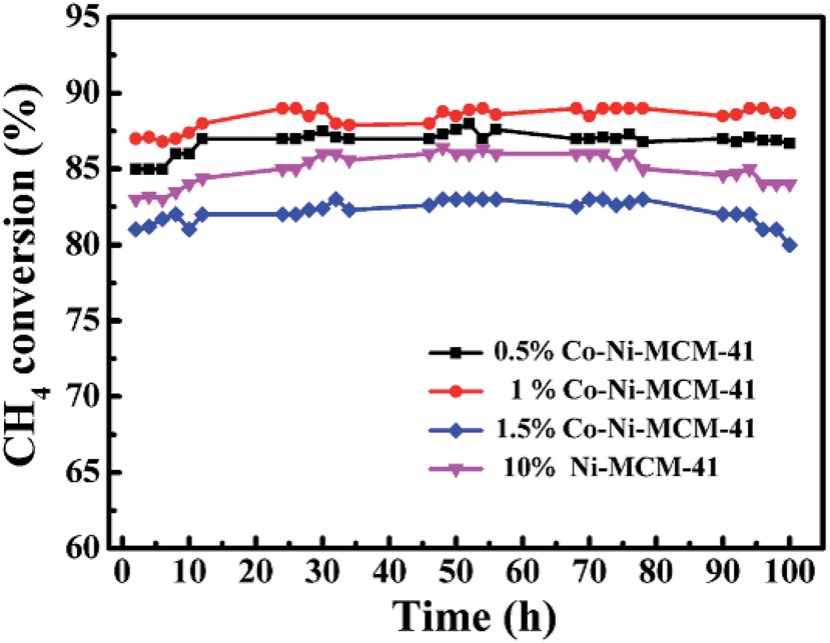
The catalytic stability comparison of the Ni–MCM-41 and Co–MCM-41 catalysts, at 750 °C, GHSV = 18 L g^−1^ h^−1^, atmospheric pressure.

The stability of Ni–MCM-41 and Co–Ni–MCM-41 catalysts were tested at 750 °C with a GHSV of 18 L g^−1^ h^−1^. As shown in Fig. 10, the CH_4_ conversion of the 0.5Co–Ni–MCM-41 catalyst and 1Co–Ni–MCM-41 catalyst reached the stability in a short time without decreasing tendency, indicating that there was no deactivation of the two catalysts. However, the Ni–MCM-41 and 1.5Co–Ni–MCM-41 catalysts showed a slight decrease in the CH_4_ conversion after the reaction of 70 h. Combining with the XRD and TGA analysis, the formation of carbon deposition on the catalyst surface leaded to the decrease of catalytic activity.

**Fig. 11:**
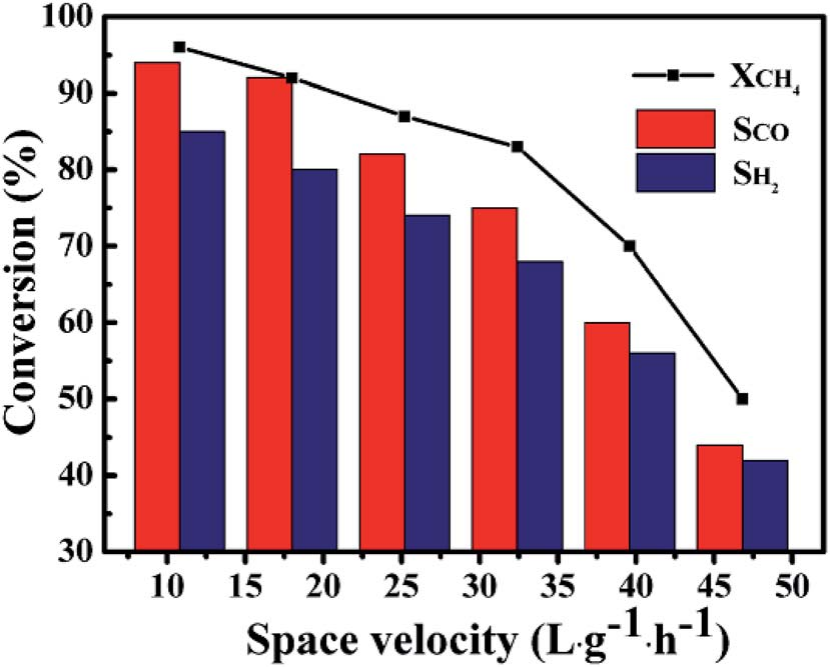
The catalytic ability as a function of GHSV over 1Co–Ni–MCM-41 catalyst at 750 °C.

Fig. 11 showed the influence of GHSV on the catalytic performance of the 1Co–Ni–MCM-41 catalyst. It was observed that the CH_4_ conversion rate and the selectivity of the product presented a slow decline trend with the rise of GHSV from 10.8 L g^−1^ h^−1^ to 32.4 L g^−1^ h^−1^. The CH_4_ conversion decreased sharply with continuous increase of GHSV, which might be caused by insufficient contact of the feed gas at the active site on the catalyst. The product selectivity presented similar trend with the CH_4_ conversion. This result might be explained by combustion reforming mechanism in the POM reaction: CH_4_ was firstly completely oxidized to CO_2_ and H_2_O, and then the remaining CH_4_ reformed with CO_2_ and H_2_O. Under fast flow rates, CH_4_ had no enough time to react with pregenerated CO_2_ and H_2_O, resulting low product selectivity.^51^

## 4. Conclusions

In this work, the well-ordered Co–Ni–MCM-41 catalysts with the assistant of Co were synthesized under direct hydrothermal condition for POM reaction. The result showed that the Ni–Co alloy formed in the channel of MCM-41 zeolite, and inhibited the formation of the NiO. With the increase of the Co content, the nickel species in the framework of zeolite decreased significantly, weakening the damage of Ni ions to the framework. The Co–Ni–MCM-41 catalysts displayed superior catalytic performance and stability than Ni–MCM-41 due to the highly dispersed Ni–Co alloy and more exposed active sites. The Co–Ni–MCM-41 catalyst remained high active after reaction of 100 h with little carbon deposition. The confinement effect of the regular pore structure and the formation of Ni–Co alloy improved the sintering and coking resistance of the catalyst. Moreover, such alloy encapsulated catalyst could also be extended to apply in other field.

## Conflicts of interest

There are no conflicts to declare.

## Supplementary Material
